# Anterior-wall and non-anterior-wall STEMIs do not differ in long-term mortality: results from the augsburg myocardial infarction registry

**DOI:** 10.3389/fcvm.2023.1306272

**Published:** 2024-01-08

**Authors:** F. Bauke, T. Schmitz, E. Harmel, P. Raake, M. Heier, J. Linseisen, A. Peters, C. Meisinger

**Affiliations:** ^1^ Epidemiology, Medical Faculty, University of Augsburg, Augsburg, Germany; ^2^ Department of Cardiology, Respiratory Medicine and Intensive Care, University Hospital Augsburg, Augsburg, Germany; ^3^ KORA Study Centre, University Hospital of Augsburg, Augsburg, Germany; ^4^ Helmholtz Zentrum München, German Research Center for Environmental Health, Institute for Epidemiology, Neuherberg, Germany; ^5^ Chair of Epidemiology, Institute for Medical Information Processing, Biometry and Epidemiology, Medical Faculty, Ludwig-Maximilians-Universität München, Munich, Germany; ^6^ German Research Center for Cardiovascular Research (DZHK e.V.), Partner Site Munich Heart Alliance, Munich, Germany

**Keywords:** acute myocardial infarction, STEMI-localization, long-term mortality, risk, non-anterior STEMI, anterior STEMI

## Abstract

**Background:**

Different ST-segment elevation myocardial infarction (STEMI) localizations go along with dissimilarities in the size of the affected myocardium, the causing coronary vessel occlusion, and the right ventricular participation. Therefore, this study aims to clarify if there is any difference in long-term survival between anterior- and non-anterior-wall STEMI.

**Methods:**

This study included 2,195 incident STEMI cases that occurred between 2009 and 2017, recorded by the population-based Augsburg Myocardial Infarction Registry, Germany. The study population comprised 1.570 men and 625 women aged 25–84 years at acute myocardial infarction. The patients were observed from the day of their first acute event with an average follow-up period of 4.3 years, (standard deviation: 3.0). Survival analyses and multivariable Cox regression analyses were performed to examine the association between infarction localizations and long-term all-cause mortality.

**Results:**

Of the 2,195 patients, 1,118 had an anterior (AWS)- and 1,077 a non-anterior-wall-STEMI (NAWS). No significant associations of the STEMI localization with long-term mortality were found. When comparing AWS with NAWS, a hazard ratio of 0.91 [95% confidence interval: 0.75–1.10] could be calculated after multivariable adjustment. In contrast to NAWS, AWS was associated with a greater <28 day mortality, less current or former smoking and higher creatine kinase-myocardial band levels (CK-MB) and went along with a higher frequency of impaired left ventricular ejection fraction (<30%).

**Conclusions:**

Despite pathophysiological differences between AWS and NAWS, and identified differences in multiple clinical characteristics, no significant differences in long-term mortality between both groups were observed.

## 1 Background

ST-segment elevation myocardial infarction (STEMI) is a potentially deadly manifestation of coronary artery disease, with mortality rates up to 14.3% in the first year after the event (1). The long-term survival of myocardial infarction patients depends on certain factors, e.g., continuation of smoking shortens survival (2). In contrast, a combination therapy with antiplatelet agents, beta-blockers, ACE-Inhibitors/Angiotensin II receptor blockers and statins is associated with an improved long-term survival (3). However, there is only limited knowledge about the associations between the admission STEMI-location and long-term mortality. STEMI can be stratified using a 12-lead electrocardiogram (ECG) into different localization types (anterior, inferior, lateral and multiple location STEMI) with reference to the anatomical localization of the ischemic myocardial area (4). An occlusion in one of the three main coronary vessels, which are right coronary artery (RCA), left anterior descending coronary artery (LAD) and left circumflex coronary artery (LCX) or its branches, leads to different STEMI localizations. The anterior-STEMI is mainly caused by ischemia of the LAD supplied myocardium (5). This type of infarction is more likely associated with severe left ventricular ejection fraction (LVEF) reduction and has a greater extent of damaged myocardium (6). Notably, 40%–50% of the left ventricle myocardium is supplied by the LAD (7). Inferior-infarction is mainly caused by vessel occlusion of the RCA, rarely of the LCX (5). In case of inferior-infarction, the right ventricle is also more likely affected (8). The infarction size itself is usually smaller than in an anterior-STEMI, especially when a smaller amount of left ventricular myocardium is affected (7). Given these pathophysiological differences, the question arises whether there is an independent association between infarction localization and long-term mortality after incident STEMI. Therefore, the present study aims to answer this question.

## 2 Material and methods

### 2.1 Study sample

The data used for this study derived from the population-based Augsburg Myocardial Infarction Registry, Germany. This registry collects data since 1984, initially as part of the MONICA project (monitoring trends and determinants in cardiovascular disease). Since the end of the MONICA project (1996), the registry operated as the KORA Myocardial Infarction Registry (9) and since 2021 as the Augsburg Myocardial Infarction Registry. The study area consists of the city of Augsburg, and two adjacent counties with a total population of approximately 680.000 inhabitants. For this study, all cases of hospitalized STEMI from 2009 to 2017 were analyzed. Patients had to have their primary residence in the study area and be aged 25–84 years. Patient data was collected by trained study nurses through interviews using standardized questionnaires during the patient's hospital stay. Medical chart reviews were carried out to provide clinical data. In this way, a great amount of data was obtained for each case of acute myocardial infarction (AMI), including cardiovascular risk factors, comorbidities, diagnostics, treatment, and sociodemographic characteristics. Information on long-term survival was kept up to date through regular follow-ups. The necessary data and death certificates were provided by the local health and registration authorities. The last mortality follow-up for this study was performed in 2019. Detailed information on data collection and definition of variables for the Augsburg Myocardial Infarction Registry can be found elsewhere (3, 10, 11). The Ethics Committee of the Bavarian Medical Association (Bayerische Landesärztekammer) approved the study procedures, and the study adhered to the Declaration of Helsinki. All patients gave written informed consent.

### 2.2 Inclusion and exclusion of cases—sample size

Only patients with incident STEMI were included without any history of previous myocardial infarction. Anterior-wall-STEMI (AWS) was defined by new ST-segment-elevations in I, aVL and/or V1–V4. In contrast, non-anterior-wall-STEMI (NAWS) was diagnosed in case of new ST-segment-elevations in II, III and aVF and/or V5/V6. At least two contiguous leads had to be affected. The cut-point for ST-segment elevations was set at ≥1 mm at the J point in all leads except V2/V3. In these two leads the elevation had to be ≥2 mm in men aged ≥40 years, respectively ≥2.5 mm in men aged <40 years to reach significance. For women included in the study, the cutpoint was set at ≥1.5 mm in V2/V3 (12).

**Figure 1:**
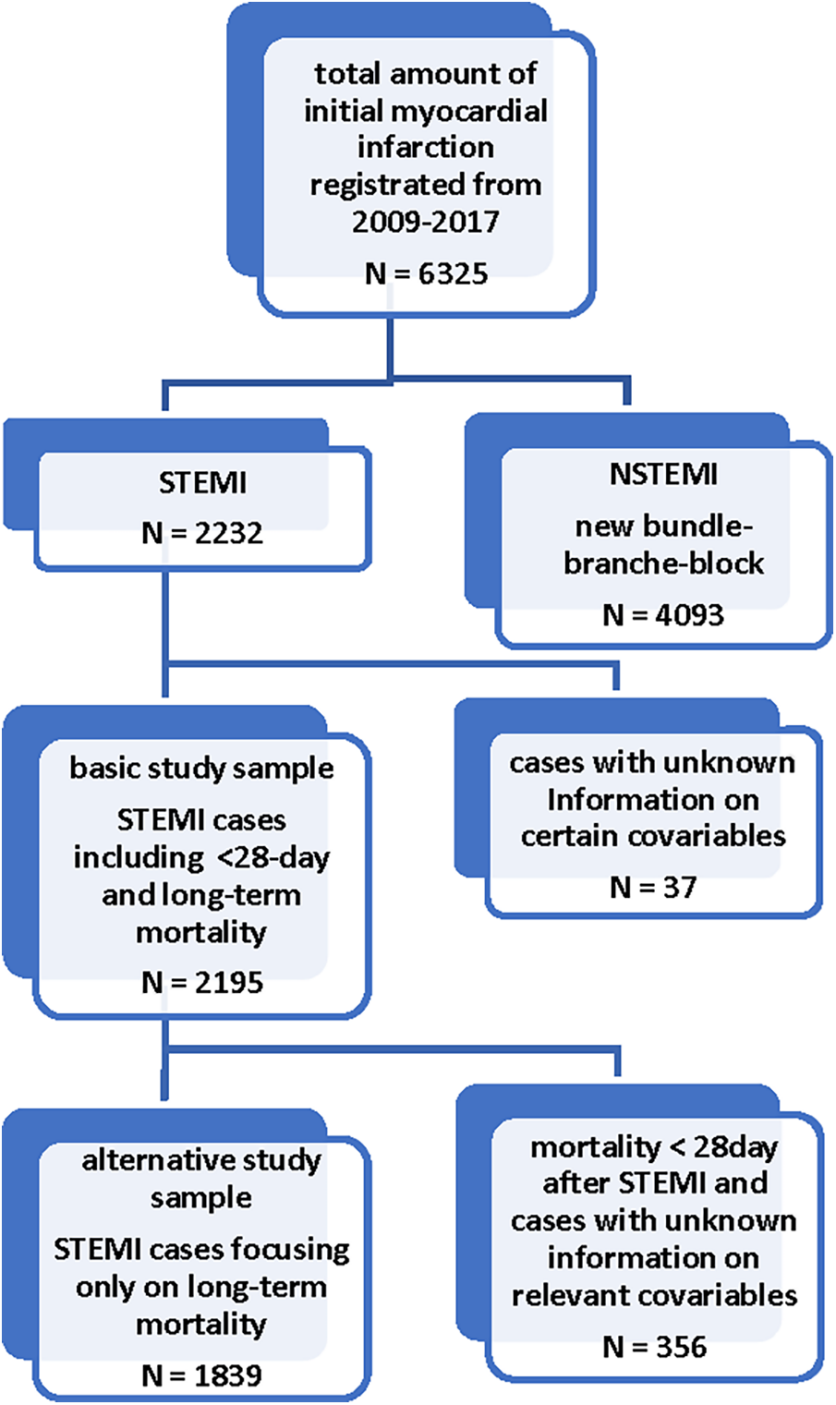
Flow-Chart for the selection of cases in the basic and alternative model.

Each STEMI was confirmed by either catheterization and/or typical chest-pain symptoms combined with a dynamic troponin elevation. Patients with bundle-branch-block and non-STEMI cases were excluded. After these exclusions, *n* = 2,232 patients remained out of a total study population of *n* = 6,325. Patients were also excluded if they had incomplete data on the following covariables: sex, age, ECG-STEMI location, arterial hypertension, diabetes mellitus, hyperlipidemia, serum creatinine, bypass surgery, percutaneous coronary intervention (PCI). Patients with concomitant AWS and NAWS were also excluded. After applying all exclusion criteria, a total of *n* = 2,195 patients were selected for statistical analysis of the basic model (AWS: *n* = 1,118 and NAWS: *n* = 1,077). It should be noted that patients with unknown information on certain variables were also used as separate categories for the statistical analysis in the basic model, including 148 cases with unknown smoking status, 124 patients without information on LVEF (left ventricular ejection fraction), 20 cases having no information on typical chest pain and 115 patients with an unknown BMI. The medication at hospital discharge was divided into two groups, whether if a combination of all four evidence-based drugs for post STEMI therapy was prescribed or it was not, e.g., in case of in-hospital mortality or due to contraindications. The combination of antiplatelet agents, beta-blockers, ACE-inhibitors/AT-II-blockers, and statins at hospital discharge was the definition for this variable. The infarction localization was determined by clinicians on the basis of the admission ECG. Infarction size was estimated from the highest creatine kinase-myocardial band (CK-MB) level. during hospitalization and was categorized as small (CK-MB: 0–150 U/L), intermediate (151–300 U/L), large (301–600 U/L) and extensively large (601 U/L). Of note, 114 patients with unknown CK-MB levels were included in the study population and kept as a separate category. Renal function was categorized by serum creatinine levels as normal (0–1.00 mg/dl), slightly impaired (1.01–1.5 mg/dl), moderately impaired (1.51–2 mg/dl) and severely impaired (≥ 2.01 mg/dl). BMI was categorized as normal weight (BMI ≤ 24.9 kg/m^2^), overweight (BMI = 25–29.9 kg/m^2^), and obese (BMI ≥ 30 kg/m^2^). In order to focus specifically on long-term mortality, an alternative statistical model was performed. The following conditions differed it from the basic analysis. Firstly an exclusion of 136 patients who died <28 days after STEMI was conducted. Secondly we excluded all cases with missing values regarding any of the other listed covariables above, except 88 cases with unknown peak CK-MB level. Therefore it contained a total of 1,839 STEMI patients. Thirdly, the medication at hospital discharge was listed in detail in this model. An overview of patient selection in both models can be found in Figure 1.

### 2.3 Outcome

The endpoint of the study was long-term all-cause mortality after the initial STEMI. The last update of the vital status was performed in 2019.

### 2.4 Statistical analysis

Baseline characteristics of the study population are given as means and standard deviations (SD) for continuous variables as well as median and interquartile-range (IQR) for creatinine. At baseline, the categorical variables are characterized using totals and percentages. Chi-squared tests were used to test for differences in categorical variables when comparing the AWS group with the NAWS group. Student's *t*-tests were performed for the continuous variables age, BMI, and follow-up time, and the Mann–Whitney *U*-test was used for peak CK-MB and creatinine levels. To estimate the effect of STEMI location on long-term mortality, a first Cox regression model was calculated using only the categorical variables AWS and NAWS without any adjustment. A second Cox regression model additionally included sex and age. Additional to the second model, the third multivariable adjusted model contained the covariables BMI, diabetes mellitus, arterial hypertension, smoking status, hyperlipidemia, typical chest-pain on admission, LVEF ≤ 30%, bypass surgery, PCI, peak CK-MB level, creatinine level, pharmacological therapy at with a combination of four evidence-based drugs at hospital discharge including antiplatelet agents, beta-blockers, ACE-inhibitors/AT-II-blockers and statins. Cox proportional hazards assumptions were assessed by visual inspection of log-minus-log transformated survival plots. No relevant violation of the proportional hazards assumption was found for any of the covariables used. Statistical analysis was performed using SPSS Version 29.0.1.0. Statistical significance was set at *p* < 0.05.

## 3 Results

**Figure 2:**
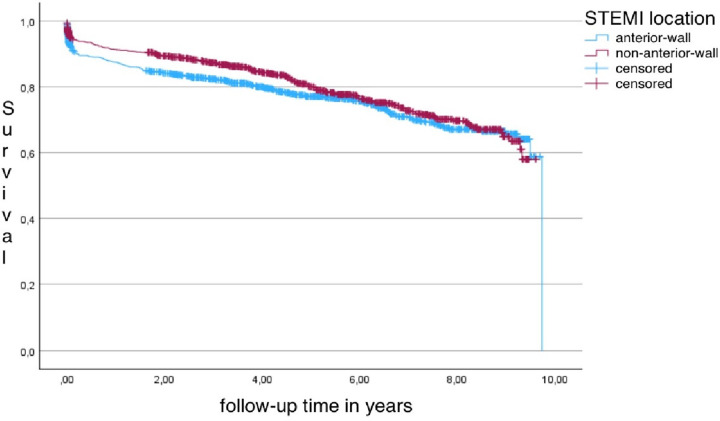
Survival curves for anterior and non-anterior-wall-STEMI in the basic model, Log-rank test: *p* = 0.142.

A total of 2,195 STEMI cases were included in the basic analysis. Of those, 484 patients (22.1%) died during the follow-up period. The mean follow-up time was 4.3 (3.0) years. Figure 2 shows the Kaplan–Meier survival curves of AWS and NAWS patients. The main objective of this study was to evaluate whether ECG infarction localization influences long-term mortality in patients with incident STEMI. The main conclusions were: (a) no significant influence of ECG-diagnosed infarction location on long-term survival in either the unadjusted Cox regression model or the sex and age adjusted model was found. Likewise, no significant effect could be shown for the fully adjusted Cox regression model. (b) AWS had a significantly higher 28-day mortality than NAWS. (c) However, AWS cases significantly differed from NAWS cases regarding mean CK-MB levels, BMI, prevalence of hyperlipidemia, use of PCI and the proportion of patients with reduced LVEF ≤ 30% or current or former smoking.

### 3.1 Baseline characteristics

Baseline characteristics of the patients by ECG-diagnosed infarction location are shown in Table 1. The AWS and NAWS groups contained comparable numbers of patients (*n* = 1,118 vs. *n* = 1,077). In general, most of the STEMI patients were men (71.5%). There was no significant difference in age or sex between the AWS and NAWS groups. AWS was significantly associated with larger infarct size (mean peak CK-MB 248.9 vs. 194.2 U/L, *p* 0.041) and a higher proportion of patients with LVEF ≤ 30%. In the NAWS group, the frequency of patients who currently or former smoked was higher compared to the AWS group. In both groups, the majority of patients received PCI revascularization. At hospital discharge, there were no significant differences between the groups regarding the treatment with the four evidence-based drugs: antiplatelets, ACE/AT-II inhibitors, beta-blockers, and statins.

**Table 1:** Baseline characteristics of included STEMI patients.

Location of STEMI	Anterior-wall	Non-anterior-wall	*p-*Values	Total sample
	*N* = 1,118 (50.9%)	*N* = 1,077 (49.1%)	–	*N* = 2,195 (100%)
General patient characteristics				
Men	799 (71.5%)	771 (71.6%)	0.950	1,570 (71.5%)
Age in years (mean/SD)	63.7 (12.4)	63.5 (11.9)	0.634	63.6 (12.1)
Follow-up time in years (mean/SD)	4.1 (3.1)	4.4 (2.9)	0.030	4.3 (3.0)
Total mortality	258 (23.1%)	226 (21.0%)	0.237	484 (22.1%)
<28-day mortality	82 (7.3%)	54 (5.0%)	0.024	136 (6.3%)
Left-ventricular EF ≤30%	143 (12.8%)	37 (3.4%)	<0.001	180 (8.2%)
Typical chest-pain	927 (82.9%)	907 (84.2%)	0.384	1,834 (83.6%)
Left-ventricular EF unknown/not measured	64 (5.7%)	60 (5.6%)	–	124 (5.6%)
Cardiovascular risk factors				
Known BMI (mean/SD)	27.3 (4.6)	27.9 (4.7)	0.007	27.6 (4.7)
BMI ≤24.9 kg/m^2^	326 (29.2%)	296 (27.5%)	0.015	622 (28.3%)
BMI 25–29.9 kg/m^2^	475 (42.5%)	441 (40.9%)	–	916 (41.7%)
BMI ≥30 kg/m^2^	248 (22.2%)	294 (27.3%)	–	542 (24.7%)
BMI unknown	69 (6.2%)	46 (4.3%)	–	115 (5.2%)
Current smoking	403 (36.0%)	438 (40.7%)	<0.001	841 (38.3%)
Former smoking	265 (23.7%)	288 (26.7%)	–	553 (25.2%)
No history of smoking	358 (32.0%)	295 (27.4%)	–	653 (29.7%)
Smoking status unknown	92 (8.2%)	56 (5.2%)	–	148 (6.7%)
Arterial hypertension	776 (69.4%)	779 (72.3%)	0.132	1,555 (70.8%)
Diabetes mellitus	297 (26.6%)	286 (26.6%)	0.996	583 (26.6%)
Hyperlipidemia	534 (47.8%)	562 (52.2%)	0.038	1,096 (49.9%)
Revascularisation therapy and hospital discharge medication				
PCI	969 (86.7%)	967 (89.8%)	0.024	1,936 (88.2%)
Bypass surgery	92 (8.2%)	86 (8.0%)	0.834	178 (8.1%)
4 evidence-based drugs (EBD)	861 (77.0%)	857 (79.6%)	0.123	1,718 (78.3%)
Unknown amount or 1–3 EBD	258 (23.1)	219 (20.3%)	–	477 (21.7%)
Laboratory values				
Known CK-MB (mean/SD) U/L	248.9 (267.6)	194.2 (162.6)	0.041	221.4 (222.8)
CK-MB 0–150 U/L	501 (44.9%)	517 (48.0%)	<0.001	1,018 (46.4%)
CK-MB 151–300 U/L	233 (20.9%)	320 (29.7%)	–	553 (25.2%)
CK-MB 301–600 U/L	205 (18.4%)	177 (16.4%)	–	382 (17.4%)
CK-MB ≥601 U/L	96 (8.6%)	31 (2.9%)	–	127 (5.8%)
CK-MB unknown	82 (7.3%)	32 (3.0%)	–	114 (5.2%)
Creatinine (median/IQR) mg/dl	1.00 (0.35)	0.99 (0.36)	0.893	1.00 (0.36)
Creatinine 0–1.00 mg/dl	571 (51.1%)	563 (52.3%)	0.865	1,134 (51.7%)
Creatinine 1.01–1.50 mg/dl	436 (39.0%)	402 (37.3%)	–	838 (38.2%)
Creatinine 1.51–2.00 mg/dl	77 (6.9%)	76 (7.1%)	–	153 (7.0%)
Creatinine ≥2.01 mg/dl	34 (3.0%)	36 (3.3%)	–	70 (3.2%)

### 3.2 Cox regression analysis

The results of the different Cox regression models are shown in Table 2. No significant association of infarction location on long-term mortality was observed in any of the Cox models. The fully adjusted model showed a hazard ratio of 0.92 [0.76–1.12], *p* = 0.409 when anterior STEMI was compared to non-anterior STEMI. Further information on hazard ratios and *p*-values of these covariables included in the multivariable model are displayed in Supplementary Table S1. The results of the alternative model can be found in Supplementary Tables S2–S4 and Figure F1.

**Table 2:** Results of the Cox regression model for anterior-wall-STEMI compared to non-anterior-wall-STEMI (reference variable) with all cause-mortality as end-point.

Cox-regression model	Unadjusted model	Adjusted for sex and age	Fully adjusted^a^
HR [95% CI]	1.14 [0.96–1.37]	1.07 [1.06–1.08]	0.91 [0.75–1.10]
*p*-value	0.142	0.220	0.305

^a^ Adjusted for: sex, age, BMI, typical chest-pain, left-ventricular EF ≤ 30%, arterial hypertension, diabetes mellitus, smoking status, hyperlipidemia, creatinine level, peak CK-MB level, bypass surgery, PCI, combination of 4 evidence-based drugs (antiplatelet agents, beta-blockers, ACE-inhibitors/AT-II-inhibitors, statins).

## 4 Discussion

The latest 2023 ESC (European Society of Cardiology) guidelines for acute coronary syndrome mention AWS as a specific risk factor for a worse short-term outcome, which agrees with our findings of a significantly higher <28-day mortality in AWS. How anterior STEMI localization affects long-term outcomes is not mentioned in these guidelines (13).

The anterior location of AMI has long been recognized as a predictor of worse outcome.

Studies conducted before the era of reperfusion therapy found higher mortality rates in patients with anterior infarction, as did more recent studies when thrombolysis or PCI was available (6, 8, 14, 15). As a result, anterior infarction has been identified as a specific risk factor in various risk stratification scores. However, the relationship between the location of the infarction, the extent of myocardial damage, and the resulting outcome for patients remains still controversial (6).

Stone et al. in 1988 investigated 471 patients with initial infarction and found a significantly worse long-term outcome for ECG-diagnosed anterior infarction compared with inferior location (8). In addition, anterior infarction was associated with a larger infarction size and a significantly lower LVEF on admission. Even when patients with comparable infarction sizes were examined, those with an anterior infarction had a significantly lower admission LVEF. Overall, cumulative cardiac mortality was higher for anterior infarction compared with inferior infarction during a mean follow-up of 30.8 months (range 0–48 months). Twenty-seven percent of patients with anterior infarction died compared to 11% in the inferior infarction group, *p *< 0.001. Comparable results were also found in a study by Hands et al. in 1986 (*n* = 789 patients), which showed significantly higher one-year mortality in anterior compared to inferior infarction (18.3% and 10.5%, respectively, *p* = 0.002) (14). They considered anterior infarction location to be an independent risk factor for survival after the first myocardial infarction, even after adjustment for infarct size. Our results contradict these previous studies conducted before the reperfusion era. One reason for the decreased influence of infarction location on long-term survival nowadays may be, that early reperfusion of acute myocardial infarction prevents remodeling, reduces infarct size, and supports left ventricular function (15).

In 1995 Welty et al. investigated the influence of location and type of AMI including 505 patients who received PCTA for post-infarction ischemia (16). Long-term follow-up data with a mean of 34.3 months were available for *n* = 440 patients (AWS: *n* = 213, NAWS: *n* = 227). It was shown that AWS compared to NAWS had a significantly higher rate of adverse outcomes (either reinfarction, death, repeated angioplasty, or coronary bypass surgery). Elsman et al. found a significantly lower LVEF in LAD-related infarction (*n* = 432) compared with non-LAD infarction (*n* = 456) at a 6-month post-AMI follow-up (43% vs. 51%, *p* < 0.001). In addition, they showed that, even after adjusting for enzymatic markers of infarct size, LAD-related infarcts were associated with a greater loss of LVEF for the same amount of necrotic myocardium compared with non-LAD-related infarcts (15). In accordance with these results, we found a significantly higher proportion of severe LVEF reduction in the AWS group, but this did not lead to an overall worse long-term survival compared to the NAWS group. In 2009, Swanson et al. studied 527 high-risk AMI patients treated with PCI followed-up for 4.8 years, which is comparable to our study (17). They found that AWS was not associated with worse long-term mortality, similar to our study. This study and our study differ in two main factors: firstly, patients with short-term mortality (<30 days survival) were included and secondly, the non-anterior infarction group was limited to more severe cases; we included all cases of NAWS without reference to severity, a fact which may explain why the prevalence of LAD related infarction was 66.6% in their study compared to 50.0% AWS in our study.

Another, more recent study of 355 patients with STEMI recanalized by primary PCI, conducted by Reindl et al. in 2019, investigated infarct localization and size using cardiac magnetic resonance imaging (cMRI) (6). In contrast to our results, they found a higher prevalence of mid-term major adverse cardiac events (MACE) for AWS compared with non-anterior infarction localization at a median follow-up of 35 months. They explained their findings by a usually higher infarction volume of left ventricular mass in anterior compared to non-anterior STEMIs (19 vs. 12%, *p* < 0.001%); the infarct location itself had no independent contribution to medium-term MACE. That study had the advantage that they could characterize the localization and size more accurately than we could, due to their use of cMRI. On the other hand, they had a smaller number of non-population-based patients in their analysis and a shorter follow-up period. So, the results are not exactly comparable to those of the present study. A recent multicentre study by Symons et al. investigated the long-term prognostic significance (median follow-up of 5.5 years) of cMRI in 810 STEMI patients. Approximately half of these patients had anterior STEMI. They found no significant effect of anterior vs. nonanterior STEMI location on their primary endpoint (death or decompensated heart failure), which occurred in 99 patients (18).

Based on our study results, we conclude that the anterior infarction location itself seems to have no significant impact on long-term survival in the era of primary PCI and highly effective evidence-based pharmacological therapy. There are risk stratification indices for STEMI that include anterior infarction location as an independent risk factor, such as the TIMI score. Furthermore, anterior infarct location is declared as a risk factor in the 2017 ESC STEMI guidelines (19, 20). On the basis of recent studies and our study results, an evaluation should be made of whether the location of anterior wall infarction should continue to be considered in long-term risk stratification for STEMI.

## 5 Strengths and limitations

A major strength of this study is the large number of STEMI patients recorded by a population-based registry with consecutive enrolment, which avoids selection bias. Another strength is the mean and maximum follow-up of 4.3 and 9.7 years, respectively, which is longer than most other comparable studies. Furthermore, we performed a statistical analysis with a large number of different covariables (cardiovascular risk factors, comorbidities, therapy, laboratory values, etc.), which allowed us to assess the long-term mortality risk of infarction localization independently of various other factors. Nevertheless, there are also some limitations. First, there was no information on the cause of death (cardiovascular event or a non-cardiovascular cause of death). Therefore, it was not possible to assess whether a particular STEMI localization was associated with a higher risk of cardiovascular mortality. In addition, the use of ECG-diagnosed infarction location and peak CK-MB to assess infarction size is not as accurate as certain cMRI techniques for assessing STEMI location and size. We also had no information on the lateral wall or right ventricular infarction, which may have impacted the results. With an age range of 25–84 years, most STEMI cases that occurred in the study area were included, but some cases may have occurred after the age of 84. Moreover, data collection for the study population started in 2009 and ended in 2017. Since 2009, there have been significant changes in the diagnosis and treatment of STEMI, which may have affected the results as well. Moreover, there was no information on ethnicity in our analysis, therefore our results may not be applicable to all ethnic groups. Finally, there may be other covariables that were not included that could have influenced our results.

## 6 Conclusions

We found no significant associations between infarction location and long-term all-cause mortality after an incident STEMI. Therefore, the initial ECG-derived location of the STEMI does not seem to be a useful tool for physicians in assessing the long-term prognosis of STEMI patients, even though the location of the infarction has been included in previous risk stratification scores. Nevertheless, AWS on average were larger and more likely to go along with severely reduced LVEF, whereas in the NAWS group the proportion of current or former smokers was higher than in the AWS group.

## Data Availability

The raw data supporting the conclusions of this article will be made available by the authors, without undue reservation.
