# Supplementary material for: Anterior-wall and non-anterior-wall STEMIs do not differ in long-term mortality: results from the augsburg myocardial infarction registry
Source: Front Cardiovasc Med. 2024 Jan 8;10:1306272. doi: 10.3389/fcvm.2023.1306272 (PMC10800510; doi:10.3389/fcvm.2023.1306272)

***Anterior-wall and non-anterior-wall STEMIs do not differ in long-term mortality: Results from the Augsburg Myocardial Infarction Registry.***

Bauke F^1,2^, Schmitz T^1^, Harmel E^2^, Raake P^2^, Heier M^3,4^, Linseisen J^1^, Peters A^4,5,6^, Meisinger C^1^

^1^ Epidemiology, Medical Faculty, University of Augsburg, Augsburg, Germany

^2^ University Hospital Augsburg, Department of Cardiology, Respiratory Medicine and Intensive Care, Augsburg, Germany

^3^ University Hospital of Augsburg, KORA Study Centre, Augsburg, Germany

^4^ Helmholtz Zentrum München, German Research Center for Environmental Health, Institute for Epidemiology, Neuherberg, Germany

^5^ Chair of Epidemiology, Institute for Medical Information Processing, Biometry and Epidemiology, Medical Faculty, Ludwig-Maximilians-Universität München, Munich Germany

^6^ German Research Center for Cardiovascular Research (DZHK e.V.), Partner Site Munich Heart Alliance, Munich Germany

Corresponding author: Ferdinand Bauke, E-Mail: ferdinand.bauke@web.de

**Table S1. Results of the fully adjusted* basic Cox Regression model**

**with all cause-mortality as an end-point.**

**patient characteristics HR [95% CI] *p-*value**

female gender 1.22 [0.99-1.51] 0.066

age per year 1.07 [1.06-1.08] <0.001

LVEF ≤ 30% (ref.) - -

LVEF > 30% 0.41 [0.32-0.53] <0.001

LVEF unknown/not measured 0.45 [0.30-0.66] <0.001

typical chest-pain (ref.) - -

no typical chest-pain 1.62 [1.29-2.03] <0.001

typical chest-pain unknown 1.26 [0.66-2.42] 0.488

**cardiovascular risk factors**

BMI 0-24.9 (ref.) - -

BMI 25-29.9 1.08 [0.85-1.37] 0.536

BMI ≥ 30 0.97 [0.73-1.29] 0.839

BMI unknown 3.05 [2.18-4.25] <0.001

current smoking (ref.) - -

former smoking 0.87 [0.67-1.14] 0.323

no history of smoking 0.59 [0.44-0.78] <0.001

smoking status unknown 2.94 [2.07-4.18] <0.001

arterial hypertension 1.09 [0.86-1.38] 0.497

diabetes mellitus 1.29 [1.05-1.58] 0.016

hyperlipidemia 0.94 [0.77-1.14] 0.534

**revascularisation therapy and hospital discharge medication**

PCI 0.89 [0.67-1.18] 0.414

bypass surgery 0.87 [0.62-1.20] 0.392

4 evidence-based drugs (EBD) 0.38 [0.31-0.47] <0.001

**laboratory values**

CK-MB 0-150U/L (ref.) - -

CK-MB 151-300U/L 1.37 [1.09-1.74] 0.008

CK-MB 301-600U/L 1.14 [0.87-1.50] 0.350

CK-MB ≥ 601U/L 1.54 [1.07-2.21] 0.021

CK-MB unknown 0.79 [0.51-1.21] 0.279

creatinine 0.00-1.00mg/dl (ref.) - -

creatinine 1.01-1.50mg/dl 1.24 [0.99-1.55] 0.058

creatinine 1.51-2.00mg/dl 1.88 [1.38-2.55] <0.001

creatinine ≥ 2.01 2.95 [2.06-4.22] <0.001

*adjusted for: sex, age, BMI, typical chest-pain, left-ventricular EF ≤ 30%,

arterial hypertension, diabetes mellitus, smoking status, hyperlipidemia, creatinine level,

peak CK-MB level, bypass surgery, PCI, combination of 4 evidence-based drugs

(antiplatelet agents, beta-blockers, ACE-inhibitors/AT-II-inhibitors, statins)

**Table S2. Baseline characteristics of included STEMI patients in the alternative model.**

**location of STEMI anterior-wall non-anterior *p-*values total sample -wall**

**N =**  **919 (50.0%) 920 (50.0%) - 1839 (100%)**

**patient characteristics**

men 675 (73.4%) 673 (73.2%) 0.885 1348 (73.3%)

age in years (mean/SD) 62.6 (12.3) 62.7 (11.8) 0.901 62.7 (12.0)

follow-up time in years (mean/SD) 4.6 (2.9) 4.7 (2.7) 0.390 4.7 (2.8)

left-ventricular EF ≤30% 91 (9.9%) 24 (2.6%) <0.001 115 (6.3%)

typical chest-pain 814 (88.6%) 808 (87.8%) 0.619 1622 (88.2%)

**cardiovascular risk factors**

BMI (mean/SD) 27.4 (4.7) 27.8 (4.7) 0.065 27.6 (4.7)

BMI ≤24.9 kg/m² 280 (30.5%) 268 (29.1%) 0.182 548 (29.8%) BMI 25-29.9 kg/m² 416 (45.3%) 394 (42.8%) - 810 (44.0%)

BMI ≥30 kg/m² 223 (24.3%) 258 (28.0%) - 481 (26.2%)

current smoking 356 (38.7%) 398 (43.3%) 0.002 754 (41.0%)

former smoking 235 (25.6%) 264 (28.7%) - 499 (27.1%)

no history of smoking 328 (35.7%) 258 (28.0%) - 586 (31.9%)

arterial hypertension 640 (69.6%) 662 (72.0%) 0.275 1302 (70.8%)

diabetes mellitus 238 (25.9%) 248 (27.0%) 0.607 486 (26.6%)

hyperlipidemia 466 (50.7%) 495 (53.8%) 0.184 961 (52.3%)

**revascularisation therapy and hospital discharge medication**

PCI 832 (90.5%) 845 (91.8%) 0.320 1677 (91.2%)

Bypass surgery 69 (7.5%) 65 (7.1%) 0.715 134 (7.3%)

beta-blockers 894 (97.3%) 879 (95.5%) 0.045 1773 (96.4%)

ACE-inhibitors 749 (81.5%) 742 (80.7%) 0.642 1491 (81.1%)

AT-II-inhibitors 99 (10.8%) 119 (12.9%) 0.152 218 (11.9%)

Ca2+-channel-inhibitors 117 (12.7%) 131 (14.2%) 0.344 248 (13.5%)

antiplatelet agents 911 (99.1%) 912 (99.1%) 0.998 1823 (99.1%)

statins 887 (96.5%) 891 (96.8%) 0.693 1778 (96.7%)

**laboratory values**

known CK-MB (mean/SD) U/L 237.2 (240.1) 189.2 (154.6) 0.058 212.7 (202.4)

CK-MB 0-150 U/L 414 (45.0%) 450 (48.9%) <0.001 864 (47.0%)

CK-MB 151-300 U/L 203 (22.1%) 278 (30.2%) - 481 (26.2%)

CK-MB 301-600 U/L 173 (18.8%) 145 (15.8%) - 318 (17.3%)

CK-MB ≥601 U/L 67 (7.3%) 21 (2.3%) - 88 (4.8%)

CK-MB unknown 62 (6.7%) 26 (2.8%) - 88 (4.8%)

creatinine (median/IQR) mg/dl 0.99 (0.33) 0.98 (0.34) 0.752 0.98 (0.34)

creatinine 0-1,00 mg/dl 489 (53.2%) 505 (54.9%) 0.262 994 (54.1%)

creatinine 1,01-1,50 mg/dl 370 (40.3%) 344 (37.4%) - 714 (38.8%)

creatinine 1,51-2,00 mg/dl 47 (5.1%) 48 (5.2%) - 95 (5.2%)

creatinine ≥ 2,01 mg/ dl 13 (1.4% ) 23 (2.5%) - 36 (2.0%)

**Table S3. Results of the Cox Regression model for anterior-wall-STEMI compared to non-anterior-wall-STEMI (reference variable) with all cause-mortality as end-point.**

**Cox-Regression model: unadjusted model adjusted for sex and age fully adjusted***

HR [95% CI] 0.94 [0.74-1.20] 0.91 [0.72-1.16] 0.89 [0.69-1.14]

*p*-value 0.630 0.439 0.361

*adjusted for: sex, age, BMI, typical chest-pain, left-ventricular EF ≤ 30%, arterial hypertension, diabetes mellitus, smoking status, hyperlipidemia, creatinine level, peak CK-MB level, bypass surgery, PCI, antiplatelet agents, beta-blockers, ACE-inhibitors, AT-II-inhibitors, Ca2+ channel blockers, statins

**Table S4. Results of the fully adjusted* alternative Cox Regression model**

**with all cause-mortality as an end-point.**

**patient characteristics HR [95% CI] *p-*value**

female gender 1.12 [0.84-1.49] 0.433

age per year 1.08 [1.06-1.10] <0.001

BMI 0-24.9 (ref.) - -

BMI 25-29.9 0.78 [0.58-1.05] 0.101

BMI ≥ 30 0.97 [0.69-1.38] 0.877

no smoking (ref.) - -

current smoking 1.92 [1.35-2.71] <0.001

former smoking 1.59 [1.17-2.17] 0.003

arterial hypertension 1.21 [0.86-1.71] 0.280

diabetes mellitus 1.48 [1.14-1.94] 0.004

hyperlipidemia 0.84 [0.66-1.08] 0.179

left-ventricular EF ≤ 30% 2.16 [1.46-3.19] <0.001

typical chest-pain 0.56 [0.41-0.76] <0.001

**revascularisation therapy and hospital discharge medication**

PCI 0.93 [0.60-1.44] 0.755

bypass surgery 1.06 [0.64-1.75] 0.824

beta-blockers 1.02 [0.56-1.84] 0.962

ACE-inhibitors 0.76 [0.50-1.16] 0.207

AT-II-inhibitors 0.72 [0.43-1.20] 0.205

Ca2+ channel-blocker 1.51 [1.10-2.07] 0.011

antiplatelet agent 0.84 [0.25-2.81] 0.777

statins 0.76 [0.42-1.37] 0.361

**laboratory values**

CK-MB 0-150U/L (ref.) - -

CK-MB 151-300U/L 1.05 [0.78-1.42] 0.730

CK-MB 301-600U/L 0.85 [0.59-1.24] 0.410

CK-MB ≥ 601U/L 1.02 [0.54-1.94] 0.942

CK-MB unknown 0.88 [0.50-1.56] 0.664

creatinine 0.00-1.00mg/dl (ref.) - -

creatinine 1.01-1.50mg/dl 1.38 [1.04-1.82] 0.026

creatinine 1.51-2.00mg/dl 1.50 [0.96-2.36] 0.075

creatinine ≥ 2.01 3.71 [2.16-6.40] <0.001

*adjusted for: sex, age, BMI, typical chest-pain, left-ventricular EF ≤ 30%,

arterial hypertension, diabetes mellitus, smoking status, hyperlipidemia, creatinine level,

peak CK-MB level, bypass surgery, PCI, antiplatelet agents, beta-blockers,

ACE-inhibitors, AT-II-inhibitors, Ca2+-channel blockers, statins,

**Figure S1. Survival curves for anterior and non-anterior-wall-STEMI in the alternative model.**

**Log-Rank test: *p* = 0.640**


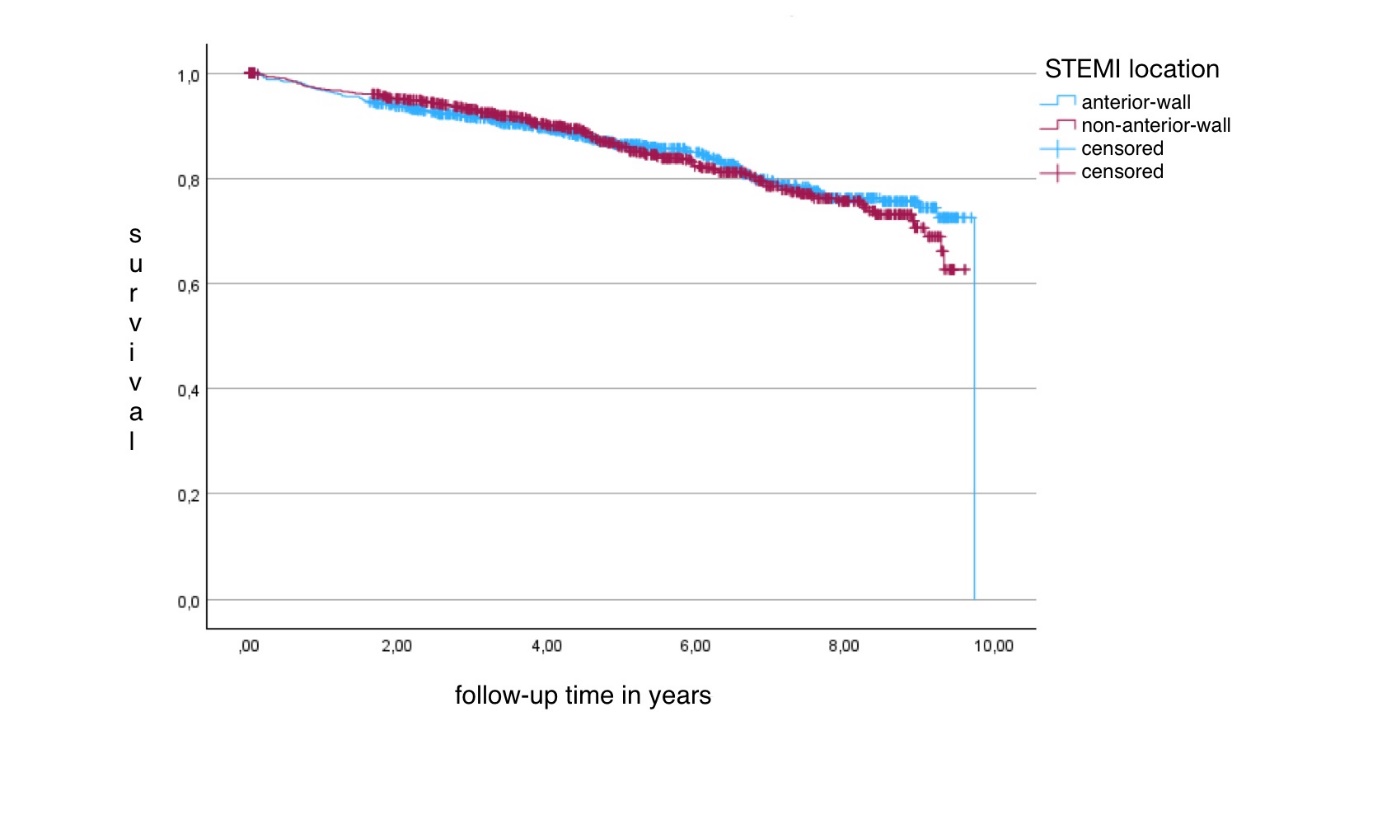

Supplement: Supplementary file 1 [file Datasheet1.docx]
